# Unique diversity of acanthothoracid placoderms (basal jawed vertebrates) in the Early Devonian of the Prague Basin, Czech Republic: A new look at *Radotina* and *Holopetalichthys*

**DOI:** 10.1371/journal.pone.0174794

**Published:** 2017-04-05

**Authors:** Valéria Vaškaninová, Per E. Ahlberg

**Affiliations:** 1 Institute of Geology and Palaeontology, Faculty of Science, Charles University, Prague, Czech Republic; 2 Subdepartment of Evolution and Development, Department of Organismal Biology, Uppsala University, Uppsala, Sweden; University of Michigan, UNITED STATES

## Abstract

The taxonomy of Early Devonian placoderm material from the Lochkovian and Pragian of the Prague basin, previously attributed to the genera *Radotina* and *Holopetalichthys*, is revised. The Pragian species *Radotina tesselata* Gross 1958 shares detailed similarities with the holotype of the Lochkovian *Radotina kosorensis* Gross 1950, which is also the holotype of the genus; the assignation of both species to *Radotina* is supported. However, the Lochkovian material previously attributed to *Radotina kosorensis* also contains two unrecognised taxa, distinguishable from *Radotina* at the generic level: these are here named *Tlamaspis* and *Sudaspis*. The disputed genus *Holopetalichthys*, synonymised with *Radotina* by some previous authors, is shown to be valid. Furthermore, whereas *Radotina*, *Tlamaspis* and *Sudaspis* can all be assigned to the group Acanthothoracii, on the basis of several features including possession of a projecting prenasal region of the endocranium, *Holopetalichthys* lacks such a region and is probably not an acanthothoracid. Skull roof patterns and other aspects of morphology vary greatly between these taxa. *Radotina* has a substantially tesselated skull roof, whereas the skull roofs of *Tlamaspis* and *Holopetalichthys* appear to lack tesserae altogether. *Tlamaspis* has an extremely elongated facial region and appears to lack a premedian plate. *Sudaspis* has a long prenasal region, but unlike *Tlamaspis* the postnasal face is not elongated. Past descriptions of the braincase of *'Radotina'* and the skull roofs of *'Radotina'* and *'Holopetalichthys'* incorporate data from more than one taxon, giving rise to spurious characterisations including an apparently extreme degree of skull roof variability. These descriptions should all be disregarded.

## Introduction

The early evolution of vertebrates has recently become a major research topic in vertebrate biology [1–8]. One of the main areas of interest is the gnathostome (jawed vertebrate) stem group, which is important from both evolutionary and phylogenetic perspectives. In evolutionary terms, the gnathostome stem group encompasses the origin of jaws and associated major changes in facial architecture [3,5,7]; in phylogenetic terms, it is a segment of the vertebrate tree whose content and topology has long been the subject of debate [9,10,11,1,2,3,4,6,7]. A key development in the understanding of this stem group has been the recognition that the placoderms (armoured jawed fishes of Silurian to Devonian age), which until recently were regarded as a clade branching off the gnathostome stem group, probably form a paraphyletic segment of that stem group [2,7,8] (but see [12]). Some groups of placoderms appear to be very primitive and close to jawless vertebrates [7] whereas others possess what were previously regarded as osteichthyan autapomorphies (notably a maxilla, premaxilla and dentary) and are probably close to the gnathostome crown-group node [6, 13].

Associated with this reinterpretation of the placoderms has been the recognition of homologies between the macromeric dermal skeleton of placoderms and osteichthyans [6], previously regarded by most workers as independently evolved ([14] but see [15] and [9]). Another Palaeozoic vertebrate group, the acanthodians (“spiny sharks”), which were previously seen as stem osteichthyans [16] or as a multiply paraphyletic array of stem gnathostomes, stem osteichthyans and stem chondrichthyans [2,4], are in the most recent analyses assigned in their entirety to the chondrichthyan stem group [6,7,8,17]. This leaves the upper part of the gnathostome stem group occupied entirely by placoderms, and gives rise to the idea of a “placoderm-osteichthyan continuum”, where osteichthyans essentially continued the gradual development of the placoderm bauplan whereas chondrichthyans departed more radically from it, *inter alia* by losing their perichondral and macromeric dermal bones [18]. Detailed studies of primitive placoderms therefore have the potential to provide crucial information about the early evolutionary steps on the path leading to our own body plan.

Acanthothoracids display many morphological features which could be considered as basal for jawed vertebrates [13,19]. Yet their morphology is poorly known except for the genus *Romundina* [7,20,21] and they occur scarcely in the fossil record (e.g. [22–30]). The Prague Basin acanthothracid collection, as reinterpreted herein, is outstanding both in diversity of species and abundance of specimens.

Early vertebrate fossils have been collected in Bohemia (part of the present day Czech Republic) for more than 150 years. Most of the specimens are now housed in public collections (Národní Muzeum (National Museum) in Prague, Czech Geological Survey and the Faculty of Science, Charles University). The collections can be considered as non-selective because almost all the rare specimens were included [31]. The best preserved remains were discovered in the period of active, especially manual quarrying in the Prague Basin (see below). The majority of the specimens described here are deposited in the Národní Muzeum in Prague (abbreviated NM in text) and most of them belong to historical collections assembled since the second half of the 19^th^ century. The most recent well-preserved specimens were collected during the second half of the 20^th^ century. Only scarce fragments have been collected in recent decades.

The Bohemian placoderms were first described by Barrande [32]. He studied several specimens from the Pragian and Emsian (Lower Devonian), among them one acanthothoracid specimen. Another specimen from the Pragian was described by von Koenen [33]. The first descriptions of acanthothoracids from the Lochkovian were accomplished by Gross [34–36] during his several stays in Prague. He described all the specimens known and accessible at that time from the Lochkovian and Pragian in detail and erected four new species (genera *Radotina* and *Kosoraspis*). The last extensive study of the Lower Devonian material from the Bohemian collections by Westoll [37] was focused on morphological terminology and phylogenetic relationships rather than taxonomy of the studied material. No other attempt to revise the Prague Basin placoderm material had been made prior to the recent studies by one of the current authors [31,38,39].

This paper examines all specimens formerly assigned to the genus *Radotina* either in published papers or in the catalogue of the NM. The published data have often been mentioned in studies on phylogenetic relationships [10] or systematics [25,40]. However, our current research on this material is uncovering a great deal of new information, both from the previously described specimens and from the extensive undescribed material in the NM collections, that not only augments but frequently contradicts the published accounts. This paper represents the first step in a complete revision of the Bohemian acanthothoracid material. In a later paper we will also be examining the enigmatic genus *Kosoraspis* [35,36], but as its taxonomic distinctness from *Radotina* is not in dispute it will not be considered here.

## Material and methods

The majority of specimens considered in this paper belong to the Národní Muzeum (NM) in Prague (the collections of the Czech Geological Survey and Charles University contain no acanthothoracid material or specimens relevant to this study). Additional specimens from the collections of the Natural History Museum, London (NHM) and Humboldt Museum für Naturkunde, Berlin (HMN) are also discussed. The specimen numbers are listed in the Systematic palaeontology section below. No permits were required for the described study, which complied with all relevant regulations.

The collections of the NM house some 300 placoderm remains; among them 25 fragments are labelled as the genus *Radotina*, three as *Holopetalichthys* and another 30 as *Macropetalichtys*. 47 samples (each with a unique catalogue number; some of them counterparts or separately numbered broken pieces), catalogued either as belonging to one of these three genera or as "Placodermi indet.", were investigated for the purposes of this revision. All the studied specimens are of Devonian age and come from two localities in the Prague Basin.

**Fig 1:**
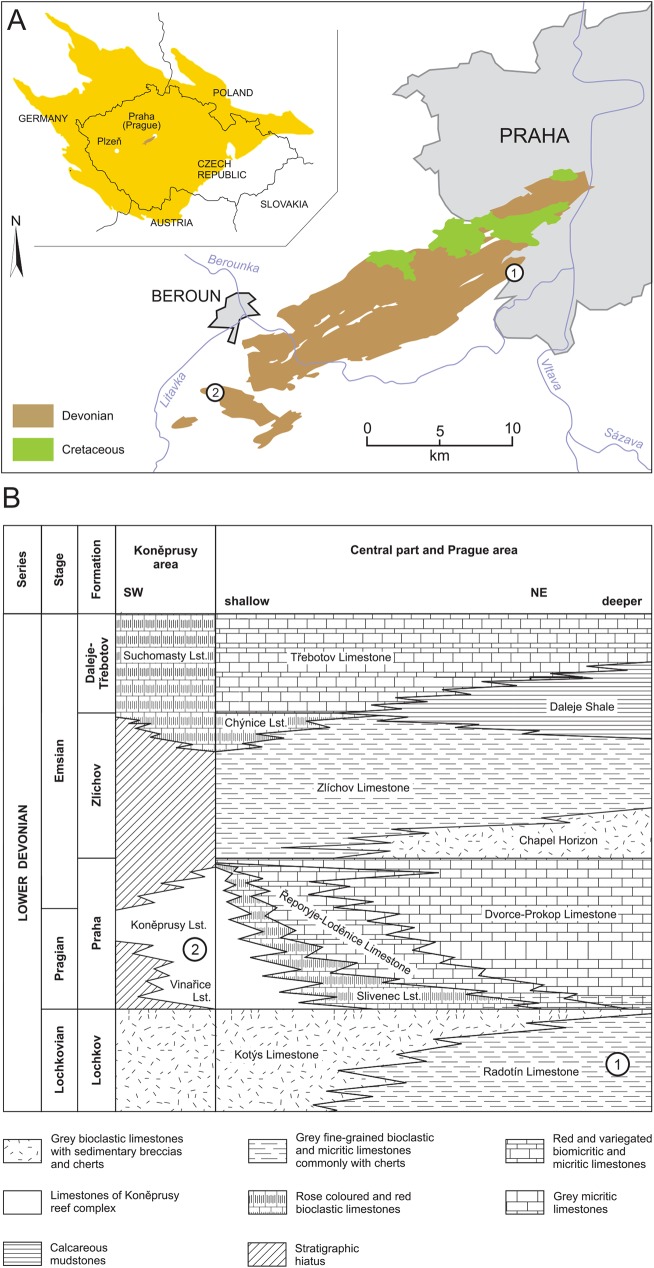
Devonian of the Prague Basin. (A) Upper left: Location of Devonian sediments (brown) in the central part of the Bohemian Massif (yellow). Right: Simplified map of the Devonian distribution. Tectonics omitted, the marine Cretaceous cover is displayed with respect to the known extent of the Devonian, younger continental units omitted; (B) Stratigraphic chart of the Lower Devonian in the Prague Basin. Numbers represent the fossil sites: 1 Černá rokle near Kosoř; 2 Koněprusy. Modified from [31] after [41].

All the specimens originally labelled *Radotina kosorensis* (most of them herein reclassified as the new genera *Tlamaspis* and *Sudaspis*) were found in the Radotín Limestone, a member of the basal Devonian (Lochkovian) Lochkov Formation (Fig 1B1) at the quarries in Černá rokle (Fig 1A1). The bony elements are very indistinct in the dark grey fine-grained matrix of the limestone when dry. Therefore all the specimens were coated with ammonium chloride before being photographed and illustrated. Some of them were mechanically prepared in the 1950s. Preparation with acetic acid (5–10%) was also tested by one of us (V.V.) on a single specimen of an isolated dermal plate, but it was unsuccessful as the bone tended to dissolve along with the matrix.

A smaller number of specimens described herein are from the Koněprusy Limestone, a member of the Lower Devonian (Pragian) Praha Formation (Fig 1B2), which is exposed in several localities near the village of Koněprusy (Fig 1A2). As the limestone is pure white and the bone elements have a reddish surface (caused by ferric oxide coating), the material could be photographed successfully without ammonium chloride coating. The Koněprusy Limestone is the only shallow water photic zone reef facies in the Prague Basin containing placoderms. The preserved specimens are distinctly smaller in size than the Lochkovian placoderms.

**Fig 2:**
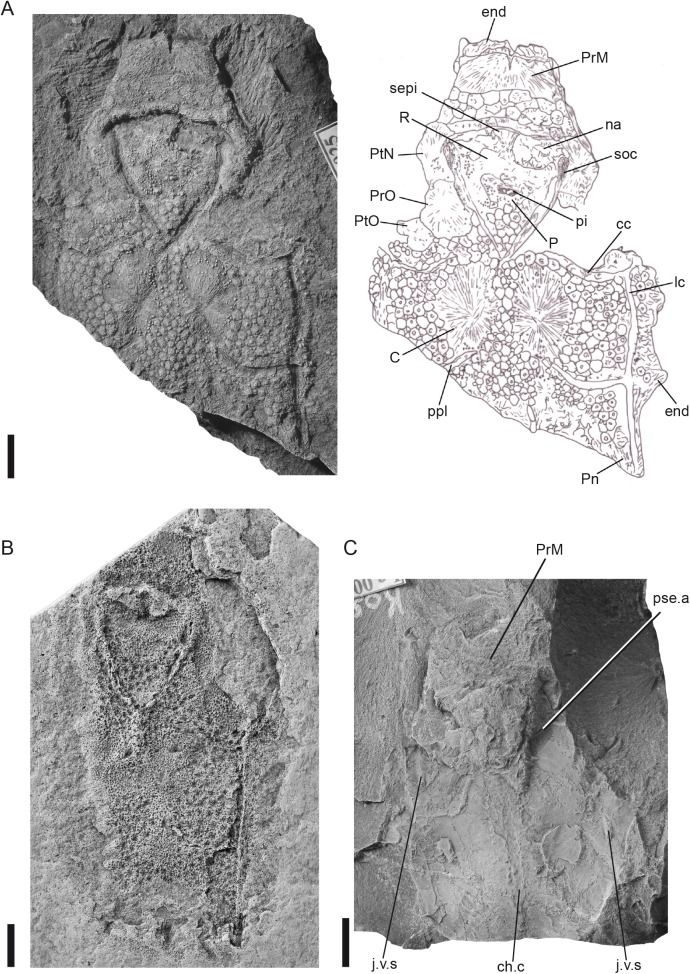
*Radotina kosorensis* Gross, 1950. (A), holotype, NM Lc 25, skull roof; (B), natural mould of external surface of skull roof, NHM P.12829; (C), anterior part of skull roof with endocranial base, NM Lc 461. Scale bars = 10 mm. Abbreviations: C central plate; cc central sensory line groove; ch.c chordal canal; end endocranium; j.v.s secondary jugular vein; lc lateral sensory line groove; na nasal openings; P pineal plate; pi pineal organ; Pn paranuchal plate; ppl posterior pitline; PrM premedian plate; PrO preorbital plate; pse.a efferent pseudobranchial artery; PtN postnasal plate; PtO postorbital plate; R rostral plate; sepi internasal septum; soc supraorbital sensory line groove.

All the specimens were studied with an Olympus SZX9 optic microscope and the drawing in Fig 2A was made with a drawing attachment Olympus SZH-DA. The surface sculpture and fine details of suitable specimens were observed under the scanning electron microscope Hitachi S-3700N and Keyence VHX-2000 digital microscope.

### Nomenclatural acts

The electronic edition of this article conforms to the requirements of the amended International Code of Zoological Nomenclature, and hence the new names contained herein are available under that Code from the electronic edition of this article. This published work and the nomenclatural acts it contains have been registered in ZooBank, the online registration system for the ICZN. The ZooBank LSIDs (Life Science Identifiers) can be resolved and the associated information viewed through any standard web browser by appending the LSID to the prefix “http://zoobank.org/”. The LSID for this publication is: urn:lsid:zoobank.org:pub:ADCEBED8-B1A1-42E6-B20A-C2DF1FEDB072. The electronic edition of this work was published in a journal with an ISSN, and has been archived and is available from the following digital repositories: PubMed Central, LOCKSS.

## Geological and palaeontological settings

The Černá rokle (= Black Gorge) quarries are situated in the south-eastern part of the Prague Basin in the Radotín Valley (Fig 1A1). Several quarries were operating there from the 19^th^ century until the 1960s. Perner [42] noted a major increase in fossil fish discoveries at the beginning of the 20^th^ century when closer contacts were established between the quarry workers and the local fossil collectors. The rocks were being quarried and processed by hand to make paving setts, and the workers were instructed and well trained in detecting precious finds. Those were then sold to the collectors, recruited from the local intellectual elite. The largest collections were amassed by A. Schubert (postmaster), F. J. Pecka (local teacher), W. Kolář (clerk) and R. Růžička (engineer); the collection of the late professor I. Chlupáč also contained a few finds acquired in his youth. The majority of these collections were donated to the NM, over the course of several decades. Crucially, important material was donated after Walter Gross had finished his studies of the NM placoderm collections in 1959, and is described here for the first time. After the quarrying ceased only collecting in debris was possible at the locality.

The outcrop represents a parastratotype of the Lochkovian/Pragian boundary and the stratotype of the boundary between the Lochkov and Prague formations [41]. The Devonian succession starts in the eastern quarries with the uppermost layers of the Lochkov Formation (Fig 1B1) composed of dark greyish platy fine-grained limestone alternating with calcareous shale facies (Radotín Limestone; [43]). Both facies are rich in invertebrate fossils [41], including bivalves (genera *Panenka*, *Neklania*, *Hercynella*, *Leiopteria*), gastropods (genera *Loxonema*, *Raphistomina*), hyoliths (*Orthotheca suavis*), tentaculites (*Paranowakia intermedia*, *P*. *geinitziana*, *Nowakia kobylica*), orthocone cephalopods, eurypterids (genus *Acutiramus*), phyllocarid crustaceans (genus *Ceratiocaris*), trilobites (genera *Spiniscutellum*, *Lochkovella*, *Lepidoproetus*, *Leonaspis*), brachiopods (genera *Howellella*, *Areostrophia*, *Plectodonta*) and graptolites (genus *Monograptus*). Vertebrate fossils include placoderms [41] and acanthodians (*Machaeracanthus bohemicus;* [44]).

The historical specimens with the lithological unit “upper part of the Koněprusy Limestone” written on the original label were most probably found at Houba´s Quarry on the southern slope of the Zlatý kůň Hill near the village of Koněprusy (Fig 1A2). Houba´s Quarry was among the first quarried in the area, exposed from 1869, and the locality is considered to be the one visited by J. Barrande [45]. The white massive limestones are either of biogenic origin, built by reef forming activities of visually recognisable sessile organisms surrounded by fine-grained micrite matrix, or coarse-grained bioclastic limestone composed of solid organic remains (Fig 1B2). They contain rich invertebrate fauna of around 500 species including stromatoporoids (genus *Actinostroma*), corals (genera *Favosites*, *Heliolites*, *Xystriphyllum*, *Pseudochonophyllum*), gastropods (genus *Platyceras*), bivalves, rostroconchs (genus *Conocardium*), trilobites (genera *Radioscutellum*, *Lioharpes*, *Gerastos*), brachiopods (genera *Sieberella*, *Hysterolites*, *Stenorhynchia*, *Eoglossinotoechia*, *Cymostrophis*, *Rynchospirina*), bryozoans (genera *Fenestella*, *Hemitrypa*, *Utropora*, *Semicoscinium*) and phyllocarid crustaceans [41]. Placoderms occur with rare specimens of large acanthodians (the genus *Machaeracanthus* may have reached 2 metres in length; [44]) and orthoconic cephalopods (genus *Ptenoceras*) in the Koněprusy Limestone.

## Systematic palaeontology

Class **Placodermi** M’Coy, 1848 [46]

Order **Acanthothoraci** Stensiö, 1944 [22]

Family **Palaeacanthaspidae** Stensiö, 1944

Genus ***Radotina*** Gross, 1950 [34]

(Figs 2 and 3)

**Fig 3:**
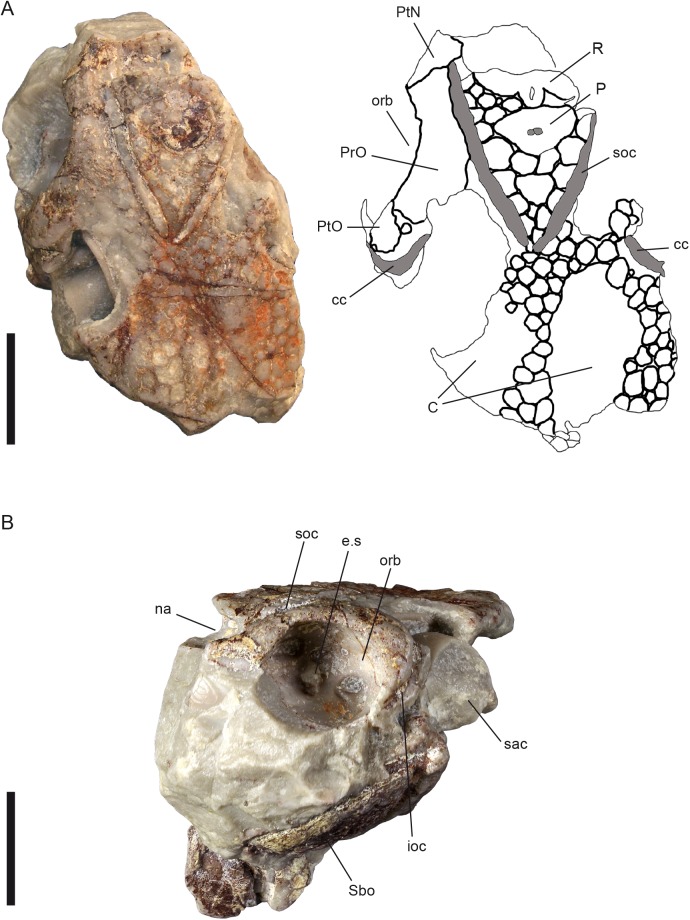
*Radotina tesselata* Gross, 1958; holotype, NM Lc 95. (A), dorsal view; (B), left lateral view; Scale bars = 10 mm. Abbreviations: C central plate; cc central sensory line groove; e.s eye stalk; ioc infraorbital sensory line groove; na nasal openings; orb orbit; P pineal plate; PrO preorbital plate; PtN postnasal plate; PtO postorbital plate; R rostral plate; sac sacculus of the inner ear; Sbo suborbital plate; soc supraorbital sensory line groove.

**Diagnosis.** Jawed vertebrate possessing a broad, flat endocranium with an anterior prenasal expansion of the trabecular region. Skull roof composed of individual small plates separated by fields of tesserae. The rostronasal capsule in dorsal position, posterior to a trapezoidal premedian area. Dorsal margin of orbit formed by three dermal plates. The rostral plate wide but anteroposteriorly short, forming a transverse bar above the dorsal margins of the nostrils. The small pineal plate, bounded by tesserae posteriorly, has a smoothly curved posterior margin, prominent lateral corners, and a transverse anterior margin. No pineal foramen. The central plates small, widely separated and of oval shape. The ornament on the dermal plates and tesserae composed of small star-shaped tubercles, each with five to six ridges that are large relative to the central body of the tubercle.

**Type species.** *Radotina kosorensis* Gross, 1950

***Radotina kosorensis* Gross**, **1950**

(Fig 2)

1950 *Radotina kosorensis* Gross; Gross [34]: pp. 113–118, fig. 1A.

1958 *Radotina kosorensis* Gross; Gross [35]: pl. 1, fig. 1–2, pl. 2, fig. 1–2; textfig. 1–2.

1959 *Radotina kosorensis* Gross; Gross [36]: pl. 3, fig. 6; textfig. 1A

1967 *Radotina kosorensis*; Westoll [37]: pp. 83–98, fig. 2A.

1969 *Radotina kosorensis* Gross; Stensiö [47], fig. 106A.

1971 *Holopetalichthys kosorensis*; Moy-Thomas & Miles [48], p. 185, fig. 8.18B.

1975 *Radotina kosorensis* Gross; Ørvig [40], pp. 45–48, 53, 66, fig. 1C.

1978 *Radotina kosorensis* Gross; Denison [25], p. 36, fig. 22B.

1984 *Radotina kosorensis* Gross; Goujet [10], pp. 228–231, fig. 8A, 12.

1993 *Radotina kosoriensis*; Lelièvre et al. [27], p. 156, fig. 7.6O.

1996 *Radotina kossorensis*; Janvier [49], p. 170, fig. 4.57H.

1998 *Holopetalichthys kosorensis* (Gross); Chlupáč [41], p. 107.

2002 *Radotina kosorensis*; Roček [50], p. 148–149, fig. 208.

2009 *Radotina kosorensis* Gross; Vaškaninová [51], p. 195.

2011 *Radotina kosorensis* Gross; Vaškaninová [38], p. 52.

**Diagnosis.**The trapezoidal prenasal part of the skull roof is formed by a short premedian plate and a posterior band of tesserae. The slightly curved central sensory line parallels the anterior margin of the central plate.

**Holotype.** Incomplete skull roof. Figured by: Gross [35], tab. 1, fig. 1, 2; tab. 2, fig. 1; textfig. 1, 2; Gross [36], tab. 3, fig. 6; textfig. 1A. Housed in the Národní Muzeum in Prague with inventory number NM Lc 25 (Fig 2A).

**Type horizon.** Lower Devonian, Lochkovian; Lochkov Formation, Radotín Limestone.

**Type locality.** Černá rokle quarries near Kosoř in Prague-Radotín.

**Material.** Skull roofs NM Lc 25, NHM P.12829 (Natural History Museum, London); endocranium NM Lc 461 (and counterpart NM Lc 462).

**Remarks.** Much of the material formerly assigned to *Radotina kosorensis* is here split off into the new genera *Tlamaspis* and *Sudaspis* (see below). These new taxa differ from *Radotina* in major aspects of cranial morphology, justifying separation at the generic level. NHM P.12829 and NM Lc 461 are, along with the holotype, the only specimens that can securely be referred to *Radotina kosorensis*. The scales attributed to *Radotina kosorensis* by Gross [36] and subsequent authors are not associated with any of these specimens. For the present they should formally be regarded as indeterminate, but their characteristic tubercle morphology suggests that they may belong to *Kosoraspis* (pers. obs. VV).

**Description.** The holotype (Fig 2A) and the so-called London specimen [35] (NHM P.12829; Fig 2B) are both incomplete skull roofs, exposed in dorsal view, that extend from the premedian plate posteriorly to the anterior part of the paranuchal plate. The holotype consists of an actual dermal skeleton whereas the London specimen is a natural mould. They do not represent the same individual.

The skull roof is composed of several dermal plates separated by fields of tesserae. In contrast to the dermal plates, the tesserae are composed of a single superficial shallow layer and are very similar to body scales [35,36]. The trapezoidal premedian plate (Fig 2A and 2C) is twice as wide as long. The ossification centre lies at its anterior border. A band of tesserae borders the posterior margin of the premedian plate and reaches towards the nostrils. The nostrils face dorsally, and the orbits are positioned posterolaterally to the nostrils. In the holotype the dorsal margins of the orbits are formed by three smaller well developed plates interpreted as the postnasal, preorbital and postorbital plates. The postorbital does not reach the posterior margin of the postorbital process and does not carry the infraorbital sensory line groove. In NHM P.12829 only two plates form the orbital margin, but a possible third plate lies between these two plates and the supraorbital lateral line groove (Fig 2B). However, it is difficult to understand the pattern as the specimen is an imperfect natural mould.

The supraorbital sensory lines emerge close to the ossification centres of the postnasal plates and continue along the mesial margins of the postnasal and preorbital plates. The supraorbital sensory lines and the nostrils outline the rostropineal area. The rostral plate is wide and anteroposteriorly short, forming a transverse bar above the dorsal margins of the nostrils. It is better preserved on the London specimen, where it touches the anterior margin of the pineal plate (Fig 2B). The small pentangular pineal plate has a smoothly curved posterior margin, prominent lateral corners, and a transverse anterior margin. There are twin recesses for the pineal and parapineal organs, positioned side by side, but no pineal foramen. The posterior part of the rostropineal area is covered by tesserae.

The central plates are oval and show slight elevations anteriorly, posteromesially and posterolaterally that appear to correspond to the underlying auditory capsules with semi-circular canals [37]. They are separated by a narrow band of tesserae and occupy the position between the central and the posterior sensory lines. Posterior to the central plates only tesserae are present. However, Gross [36] noted some faint radiating striae resembling the middle layer of a bony plate in the posterolateral corner of the holotype near the main sensory line canal (Fig 2A). We interpret this as the anterior end of the paranuchal plate. (Note that we refer to the plate that forms the posterolateral corner of the skull roof as a "paranuchal" or "posterior paranuchal" in different taxa, depending on whether a bone identified as an "anterior paranuchal" is present. This is somewhat unsatisfactory but follows standard usage in the placoderm literature [25].) The anterior part of the skull roof, from the premedian plate as far back as the centrals, has proportions very similar to *Romundina stellina* [21]; the only noteworthy difference is that *Radotina kosorensis* has smaller orbits. However, the posterior part of the skull roof appears to be considerably longer in *R*. *kosorensis* than in *Romundina stellina*.

The ornament on the dermal plates and tesserae is composed of small star-shaped tubercles, each with five to six ridges that are large relative to the central body of the tubercle. On the central plates the tubercles are restricted to the marginal areas, where they are arranged concentrically. The tesserae are tiny plates, each carrying an individual tubercle or a small group.

The sensory lines have the form of deep, wide-open grooves. They do not cross the ossification centres of the bony plates. Their pattern is most clearly seen on the holotype (Fig 2A). The supraorbital sensory lines are deep, gradually widening in the anterior part. The anterior part of the main sensory line and the central sensory lines are separated from the postorbital plate by a band of tesserae. The central sensory line is gently S-shaped and runs towards the connection of the supraorbital grooves. The posterior sensory line curves anteriorly, ending just posterior to the central plate. Where a sensory line groove passes through a tesserate area, it passes between rather than through individual tesserae, and the pattern of the tesselation is somewhat disturbed. In a few areas, notably on the main sensory line posterior to its junction with the posterior sensory line, a separate lining for the sensory canal itself is preserved within the groove. This lining is composed of very thin bone with fine transverse striations and presumably lay within the membranous wall of the sensory line canal.

The median part of the endocranial base is preserved on the specimen NM Lc 461 (and its counterpart NM Lc 462; Fig 2C). This specimen does not show the dermal skull roof, but is assigned to *Radotina kosorensis* because of the similar proportions of the premedian region. As is frequently the case in specimens from Černá rokle, the split between slab and counterslab does not follow a single plane; in the middle and posterior parts, the split mainly follows the internal face of the perichondral bone floor of the braincase (though it jumps to the floor of the sacculus on both sides), but anteriorly it rises through the telencephalic region of the braincase before entering the basal layer of the dermal bone of the premedian region. The endocranium has a roughly rhombic shape with markedly projecting postorbital processes. The most striking feature of the endocranium is the imprint of the notochordal canal that is surrounded by a vascular web and several openings. The notochord reaches far forward, ending anterior to the postorbital processes. Other prominent features are traces of canals on the lateral margins of the endocranial base, possibly branches of the jugular vein, and anteriorly a curved transverse groove for the pseudobranchial artery. The telencephalic recesses and olfactory nerve canals can be seen in section.

***Radotina tesselata* Gross**, **1958**

(Fig 3)

1950 *Radotina* sp. Gross; Gross [34], pp. 113–118, fig. 1C.

1958 *Radotina tesselata* n. sp.; Gross [35], pl. 2, fig. 3, 4, pl. 3, fig. 1–4; textfig. 3–5.

1959 *Radotina tesselata;* Gross [36], pp. 3, 4, 9.

1967 *Radotina tesselata;* Westoll [37], pp. 83–85, 88.

1969 *Radotina tesselata* Gross; Stensiö [47], fig. 51, 104 B.

1975 *Radotina tesselata* Gross; Ørvig [40], pp. 45, 48, 63, 64, 66.

1978 *Radotina tesselata* Gross; Denison [25], p. 36.

1984 *Radotina tesselata*; Goujet [10], pp. 230, 231, fig. 12.

1986 *Radotina tesselata*; Young [11], p. 17, fig. 7C.

2009 *Radotina tesselata* Gross; Vaškaninová [51], p. 195.

2011 *Radotina tesselata* Gross; Vaškaninová [38], p. 52

**Diagnosis.** The only known specimen is much smaller than *R*. *kosorensis*. The central sensory line canals point anteriorly and do not parallel the margins of the central plates. The supraorbital canals form a more acute angle than in *R*. *kosorensis*. Tesserae in the rostronasal area bigger than tesserae on the rest of the skull roof.

**Holotype and only specimen.** Anterior part of the cranium, missing the prenasal region. Figured by Gross [35], pl. 2, fig. 3, 4; pl. 3, fig. 1–4; textfig. 3–5. Housed in the Národní Muzeum in Prague with inventory number NM Lc 95 (Fig 3).

**Type horizon.** Lower Devonian, Pragian; Praha Formation, Koněprusy Limestone.

**Type locality.** Koněprusy near Beroun, south-east of Prague, probably the area of the present day Houba´s Quarry.

**Remarks.** Gross [35] describes one specimen of this species only–the holotype. It is a three-dimensional cranium with badly preserved ornamentation of the skull roof plates. The prenasal region and the area posterior to the centrals are missing. Both orbits and nostrils were prepared by a technician of W. Gross. Later a part of the endocranial base was prepared away by an unknown technician to expose the posterior part of the perichondrally lined brain cavity and ventral parts of the inner ear cavities. Although this specimen is much smaller than the holotype and other specimens of *Radotina kosorensis*, it has a partly ossified braincase (missing only the premedian region) and is thus presumably close to adult size.

**Description.** The pattern of the skull roof plates is similar to *R*. *kosorensis* (Fig 3A), although the premedian plate is unknown. Postnasal, preorbital and postorbital plates border the dorsal margin of the orbits. The supraorbital sensory line emerges at the posteromesial margin of the small postnasal plate and continues posteriorly along the mesial margin of the large preorbital plate. The postorbital plate is small and does not carry any sensory line canal. The pineal plate is small with prominent anterolateral corners. It covers both recesses for the pineal and parapineal organs and does not display a pineal foramen. The rest of the rostropineal area is covered by tesserae substantially larger than the ones covering the rest of the skull roof. The outline of the rostral plate is unclear, but it is evident that tesserae are present also anterior to the pineal plate. The central plates are oval with no sensory lines crossing. They are separated by tesserae.

The supraorbital sensory lines form a more acute angle (approx. 60°) than in *R*. *kosorensis* (70° on the holotype). The infraorbital sensory line groove runs along the posteroventral margin of the postorbital plate. A similar groove on the ventral margin of the postnasal plate may represent its anterior continuation. The central sensory line parallels the orbit and the posteromesial margin of the postorbital plate, pointing anteromesially towards the spot where the supraorbital sensory line touches the margin of the preorbital plate. This contrasts with the condition in *R*. *kosorensis* (see above), where the central sensory line is transverse and gently S-curved. All sensory lines are developed as deep and wide canals. In a few areas the grooves are covered by a thin bony lamina representing the wall of the canal itself, as described for *R*. *kosorensis*.

Two dermal plates are clearly visible on the left lateral side of the holotype (Fig 3B). Gross [35] described them tentatively as "mandibulare". A slightly curved sensory line is detectable on both plates. We interpret the larger plate as the left suborbital, which is supported by its position relative to the orbit. The second plate could be a broken and displaced right suborbital plate.

The nasal cavities, which face anteriorly, are placed medially to the anterior ends of the supraorbital sensory lines. They have an oval shape and were separated by a thin, incompletely closed septum internasale ([35]; unpreserved at present). The posterior wall of each cavity is formed by a lamina cribrosa pierced by numerous perichondrally lined canals for branches of the olfactory nerve. Imprints of blood vessels supplying the olfactory organ are preserved on the capsulae walls.

The orbits, which are relatively larger than in *Radotina kosorensis*, occupy an anterior lateral position, posterior to the nostrils. Their inner space is wider than the opening and reaches beyond it towards the area of the central sensory lines. Medially the orbit reaches the level of the supraorbital sensory lines. The internal surface of the orbits shows apertures for nerves and blood vessels, as well as a small triangular eye stalk (Fig 3B). A large opening for the optic nerve lies anterior to the eye stalk. The aperture for the jugular vein is situated in the posterolateral area of the orbit.

The otic part of the cranial cavity in the posterior ventral part of the holotype (posterior to the orbits and the dislocated suborbital plate) has been prepared by an unknown technician. The ventral and partly lateral sides of the cavities lined by a thin perichondral lamina are exposed. Anteriorly the remains of a narrow endocranial base—the medullar part of the endocranial cavity—are visible. The most prominent structures are the sacculi with the semicircular canals.

**Genus *Tlamaspis* gen. nov.** *urn:lsid:zoobank.org:act:E4B78421-D50B-4D38-BDB3-16B34C37E489*

(Figs 4–6)

**Fig 4:**
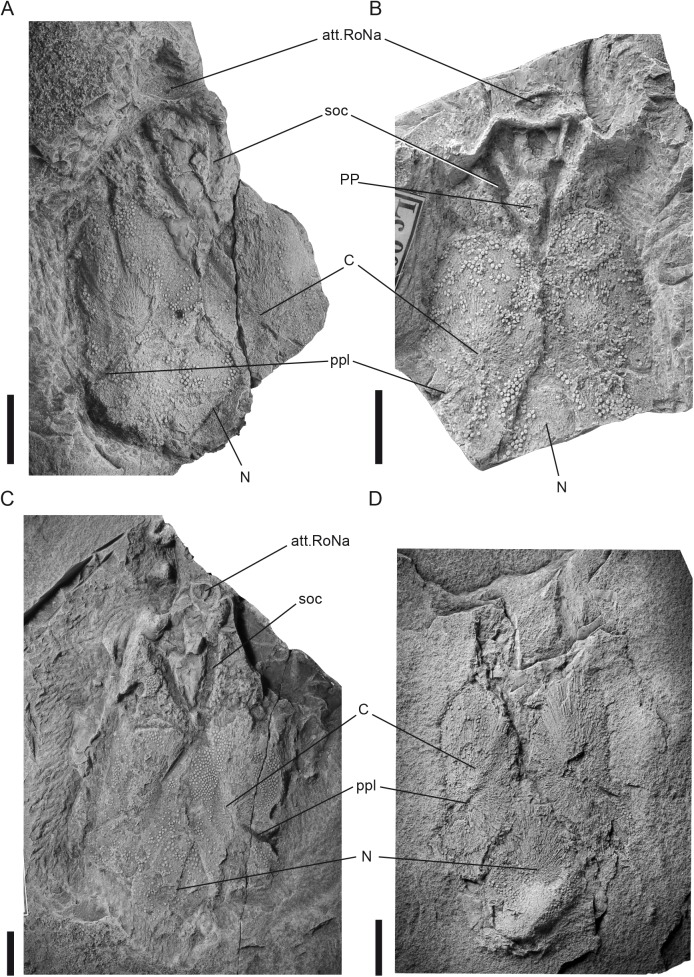
*Tlamaspis inopinatus* gen. et sp. nov. Skull roofs. (A), holotype, NM Lc 34, skull roof attached to endocranium; (B), central part of skull roof, NM Lc 29; (C), nearly complete skull roof, NM Lc 22; (D), skull roof, NM Lc 491. Scale bars = 10 mm. Abbreviations: att.RoNa attachment for rostronasal capsule; C central plate; N nuchal plate; PP postpineal plate; ppl posterior pitline; soc supraorbital sensory line groove.

**Fig 5:**
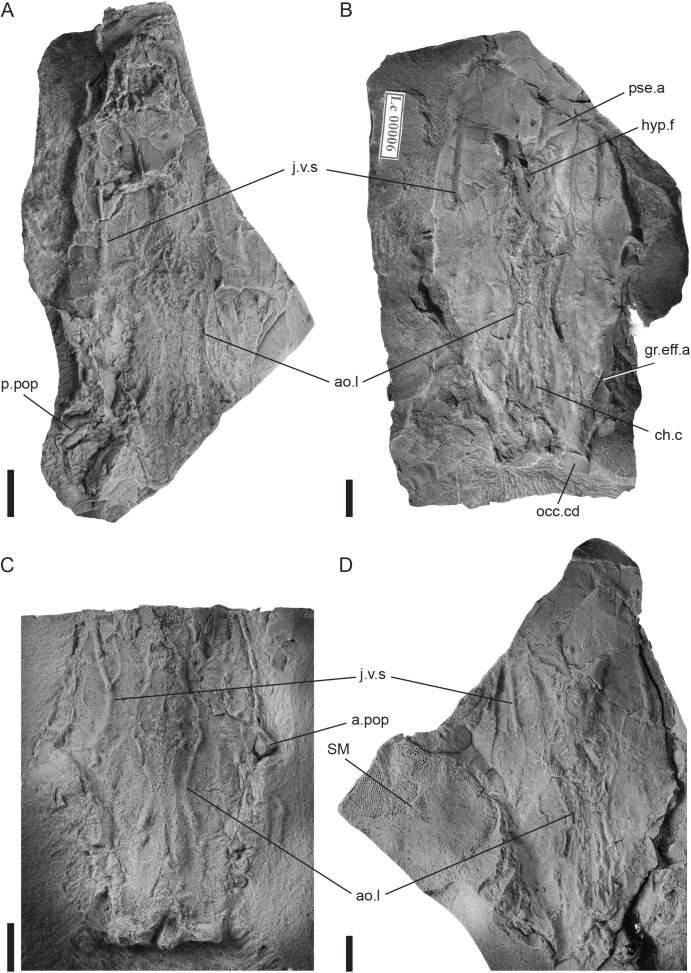
*Tlamaspis inopinatus* gen. et sp. nov. Endocrania. (A), holotype, NM Lc 35, endocranial base with rostronasal region; (B), endocranial base (counterpart of NM Lc 7), NM Lc 6; (C), posterior part of endocranial base, NM Lc 9; (D), endocranial base with rostronasal region and inner cast of submarginal plate, NM Lc 7. Scale bars = 10 mm. Abbreviations: ao.l lateral aorta; a.pop anterior postorbital process; ch.c chordal canal; gr.eff.a groove for the common efferent artery; hyp.f hypophyseal fenestra; j.v.s secondary jugular vein; occ.cd occipital condyle; p.pop posterior postorbital process; pse.a efferent pseudobranchial artery; SM submarginal plate.

**Fig 6:**
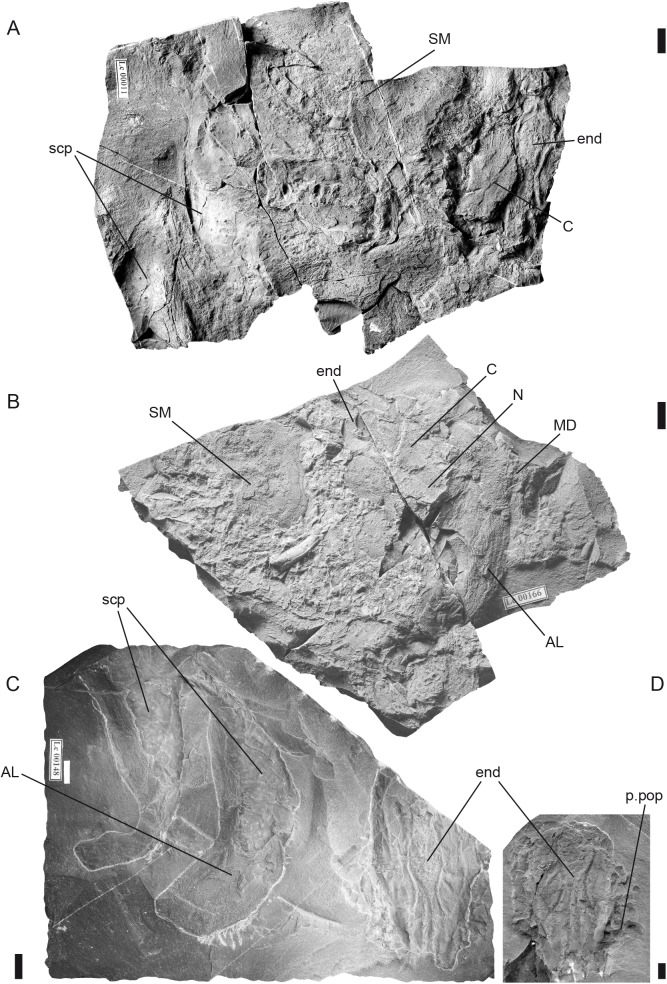
*Tlamaspis inopinatus* gen. et sp. nov. Associated skull roofs, cheek and thoracic plates. (A), NM Lc 11; (B), NM Lc 166; (C), posterior part of endocranial base associated with both shoulder girdles, NM Lc 148; (D), posterior part of endocranial base (counterpart of NM Lc 148), NM Lc 467. Scale bars = 10 mm. Abbreviations: AL anterior lateral plate; C central plate; end endocranium; MD median dorsal plate; N nuchal plate; p.pop posterior postorbital process; scp scapulocoracoid; SM submarginal plate.

**Diagnosis.** Jawed vertebrate possessing a broad, flat endocranium with an anterior prenasal expansion of the trabecular region. Prenasal area very long, occupying at least one quarter of the whole length of the endocranium. Premedian area of the endocranium contains an elaborate canal network. Glenoid region of endocranium broad. Grooves of lateral aortae on ventral wall of endocranium deeply impressed, lyre-shaped, continuing forward to hypophysial fossa. Endocranial surface rugose between grooves of lateral aortae. Grooves of secondary jugular veins deeply incised, narrow, s-curved, main mesial branch attached posterior to crossing of efferent pseudobranchial artery. Dermal skull roof contains one pair of large oval central plates, meeting in the midline anteriorly but posteriorly separated by the nuchal plate. Premedian plate absent. Posterior margin of dermal skull roof forms a projecting midline point created by the nuchal. Dermal ornament consists of small, rounded tubercles with eight to nine moderately prominent ridges.

**Derivation of name.** After the Czech/Slovak word for large mouth–“tlama”, and the Greek “aspis” (shield).

**Type and only known species.** *Tlamaspis inopinatus* sp. nov.

**Remarks**. Most of the specimens we redescribe as a new genus *Tlamaspis* were originally included in *Radotina kosorensis* by Gross [36]. In fact they differ from this taxon in numerous respects including the absence of a premedian plate, very large central plates that contact each other and the nuchal plate without intervening tesserae, tubercles of a distinctive morphology, and an elongate prenasal region. Previous authors [35,36,37,10] have explained the absence of tesserae in the *Tlamaspis* specimens, in contrast to their presence in *Radotina kosorensis*, either as individual variation or as due to taphonomic processes. However, taphonomic loss can be ruled out, because the marginal areas of the central and nuchal plates of *Tlamaspis* (which, under the taphonomic hypothesis, would in the holotype of *Radotina kosorensis* lie underneath fields of tesserae) are covered with firmly attached tubercles and have clearly not been exposed by post mortem stripping-off of tesserae. Individual variation is difficult to formally disprove without a statistically significant morphometric sample, but we note that all the *Tlamaspis* specimens are consistently similar, and different from *Radotina kosorensis*, with respect to the characters mentioned above.

***Tlamaspis inopinatus* sp. nov.** *urn:lsid:zoobank.org:act:874093B8-ED0C-4133-8418-1DCA118E945E*

(Figs 4–6)

1959 *Radotina kosorensis* Gross [36]; Gross, pl. 1; pl. 2, fig. 1–5; pl. 3, fig, 1–4, 7; pl. 4, fig. 3; pl. 5, fig. 3–4; textfig. 1B-E; 2A, D-E; 3; 4A;

1969 *Radotina kosorensis* Gross [47]; Stensiö, fig. 9B, 105A, 193A.

1975 *Radotina kosorensis* Gross; Ørvig [40], fig. 1B.

1978 *Radotina kosorensis* Gross; Denison [25], fig. 2E, 6E.

2002 *Radotina kosorensis*; Roček [50], fig. 205.

**Diagnosis.** As for genus.

**Derivation of name.** From Latin *unexpected*.

**Holotype.** Endocranium with medial skull roof plates. Figured by Gross [36] tab. 2, fig. 1,3, textfig. 1B. Housed in the Národní Muzeum in Prague with inventory number NM Lc 34 (and counterpart Lc 35; Figs 4A and 5A).

**Type horizon.** Lower Devonian, Lochkovian; Lochkov Formation, Radotín Limestone.

**Type locality.** Černá rokle near Kosoř in Prague-Radotín.

**Material.** endocrania NM Lc 6 (and counterpart NM Lc 7), NM Lc 8 (and counterpart NM Lc 9), NM Lc 10, NM Lc 34 (and counterpart NM Lc 35); endocrania with attached skull roof plates NM Lc 22 (and counterpart NM Lc 23), NM Lc 29, NM Lc 491; endocranium associated to shoulder girdle NM Lc 148 (and counterparts NM Lc 467 and NM Lc 471); endocrania associated to shoulder girdle, cheek and other dermal plates and bony elements NM Lc 11 (and counterparts NM Lc 12 and NM Lc 13), NM Lc 165 and NM Lc 168 (and counterpart NM Lc 489), NM Lc 166 (and counterpart NM Lc 167); shoulder girdle NM Lc 19 (and counterpart NM Lc 20), NM Lc 463 (and counterpart NM Lc 464); shoulder girdle associated with scales NM Lc 174 (and counterpart NM Lc 175), NM Lc 197.

**Description.** The skull roof is dominated by a pair of large, oval central plates, which meet in the midline but are separated posteriorly by a teardrop-shaped nuchal plate (NM Lc 22, NM Lc 29, NM Lc 34, NM Lc 491; Fig 4). Each central plate has a distinct, raised area in the middle, with a strongly radial texture and few or no tubercles. The nuchal plate is large, tapering anteriorly, and has a posterior oval projection that covers the underlying occipital process of the endocranium. The central and nuchal plates all have well-defined margins, but they are not quite as tightly joined as the bones of a typical sutured skull roof (see for example *Holopetalichthys*, below). Tesserae are absent between these plates. No premedian plate is present in any specimen, even though the premedian region is preserved; we infer that it is genuinely absent. The rostronasal capsule is not preserved, but its attachment area on the braincase is small. A small rhombic postpineal plate situated between the supraorbital sensory lines is known only in one specimen (NM Lc 29; Fig 4B). The supraorbital sensory lines are deep, and usually leave impressions on the dorsal side of the endocranium (Fig 4A–4C). They form a narrow V shape and meet behind the small postpineal plate. The central plates carry anteromedially directed posterior sensory lines running in the direction of the ossification centres, as well as middle and anterior pit lines. The remaining sensory lines and the openings for the endolymphatic ducts are unknown. The dermal ornament consists of small, rounded tubercles with eight to nine moderately prominent ridges.

On specimen NM Lc 7 (Fig 5D) the inner surface of a relatively large submarginal plate is exposed adjacent to the postorbital process. Similar large oval submarginal plates are present in specimens NM Lc 11–13, NM Lc 165 and NM Lc 166 (Fig 6A and 6B). Anteriorly and posteriorly to the submarginal plate of NM Lc 7, and laterally to the endocranium, lie two other damaged plates that are difficult to identify. The posterior one could be the dorsal margin of the anterior lateral plate.

Specimens NM Lc 11–13, NM Lc 165 and NM Lc 166 (Fig 6A and 6B) display a cluster of tubular bony endoskeletal elements in various states of preservation in the area between the endocranium and the suborbital plate. We interpret them as elements of the visceral skeleton, most probably the gill arches.

The thoracic shield of *Tlamaspis* is incompletely known. The most frequently preserved shoulder girdle element is the anterior lateral (AL) plate with the perichondrally ossified scapulocoracoid firmly attached to its mesial side, which is seen more or less clearly in NM Lc 11, NM LC 148 and NM Lc 166 (Fig 6). Gross [36] was unsure about the taxonomic identity of the few isolated anterior lateral plates from Černá rokle that he was able to study. Their determination to the genus *Tlamaspis* is now confirmed by specimen NM Lc 148 (Fig 6C), which was unknown to him. The anterior lateral plate is narrow, tall and crescent shaped with the postbranchial lamina situated on the concave margin. Anteroventrally it appears to taper to a point. In overall appearance it somewhat resembles an early osteichthyan cleithrum, such as that of an onychodont [52] or porolepiform [53]. The tubercles on the postbranchial lamina are fine and dense. The ossification centre lies in the anterior area of the plate. The shape of the scapulocoracoid follows the shape of the AL plate. The scapulocoracoid is composed of an anterior coracoid process and a dorsal scapular blade (Fig 6A and 6C). Numerous imprints and openings for blood vessels and nerves are clearly visible on the surface. It is possible to recognize dorsal and ventral groups ([47], figure 193A).

In NM Lc 166 the AL, which is preserved in approximate life position relative to the braincase (Fig 6B), is overlapped dorsally by the median dorsal plate (MD). This positional relationship is likely to be slightly disturbed, as it leaves no room for the dorsolateral thoracic plates that would be expected to carry the main lateral line canal of the trunk, separating the AL from the MD. The median dorsal plate has anterolateral projections that give the bone a butterfly-like outline (Fig 6B). The dorsal spine is broken off. The remaining plates of the thoracic shield are as yet unknown or unrecognisable.

The most abundant remains of *T*. *inopinatus* are the dorsoventrally compressed, perichondrally ossified endocrania. Most commonly the rock slab containing the specimen has split horizontally along the ventral face of the endocranium, with the crack sometimes running above and sometimes below the thin ventral layer of perichondral bone, so that the bone is divided in patches between slab and counterslab. Curiously, in all specimens examined to date the crack rises dorsally in the prenasal region, so that at least part of this region ends up attached to the counterslab (Fig 5B and 5D). The dorsal surface of the endocranium is largely covered by the central and nuchal plates of the skull roof (see above), except anteriorly where the rostronasal-prenasal region is exposed. (Figs 4B, 4C and 5A). In ventral view, the outline of the endocranium is clearly visible, but the actual lateral walls are difficult to reconstruct because of the strong dorsoventral compression of the specimens.

The endocranium is relatively slender. The facial region, from the postorbital processes to the anterior margin of the endocranium, is remarkably long. It comprises more than 50% of the length of the endocranium, whereas the corresponding proportion in *Romundina stellina* is approximately 42% (measurements taken from endocranium in [21]). The pre- and postnasal parts of the facial region appear in proportional terms to be equally elongated relative to the condition in *Radotina kosorensis* or *Romundina stellina*. In other words, the facial region of *Tlamaspis* appears isometrically "stretched" compared to these taxa. In general morphological terms the prenasal area is reminiscent of that in *Romundina* [7,21], with broad suborbital shelves separated in the midline by a raised, anteriorly flaring region that carries the attachment for the rostronasal capsule posteriorly and the premedian area anteriorly (Figs 4B, 4C and 5A). Unlike in *Romundina* and *Radotina*, there is no premedian plate in *Tlamaspis*, but a distinct premedian area can be recognised in the region where a premedian plate would be expected (Fig 5A). The dorsal surface of the endocranium lacks external perichondral ossification in this area, exposing a complex internal network of perichondrally lined canals.

The endocranium is widest at the level of the short anterior postorbital process (Fig 5B and 5C). The posterior postorbital process is robust but appears to be relatively short (Figs 5A and 6D). Posterior to this process, the occipital region is proportionately much shorter than in *Romundina* [21] and the condyles are strikingly wide. Mesial to the posterior postorbital process, the ventral surface of the endocranium carries a wide but not very deep longitudinal groove, bounded mesially by a ridge (Fig 5B, gr.eff.a). A similar groove in *Romundina* has been interpreted as housing a common efferent branchial artery [21].

The hypophyseal opening is slightly posterior to the rostronasal area. Anterior to the hypophysial foramen and mesial to the pseudobranchial arteries two smaller openings are situated. These can be matched in *Romundina* ([20], figures 2B,3D), where they communicate with the dorsal surface of the rostronasal area through two perichondrally lined canals that pass vertically through the cartilage of the endocranium. Their function remains uncertain.

Deeply impressed imprints of paired blood vessels are visible on the ventral surfaces of all the endocrania. The grooves for the efferent pseudobranchial arteries (Fig 5B, pse.a) originate anterior to the hypophysial fossa and follow a curved transverse trajectory similar to that in *Romundina* [7, 21]. Posteriorly there are lyre-shaped imprints of the lateral aortae. The endocranial surface between the grooves of the lateral aortae is rugose. The grooves of the secondary jugular veins (Fig 5, j.v.s) are deeply incised, narrow, and s-curved, and their main mesial branch is attached posterior to the crossing with the efferent pseudobranchial artery. The course of the notochord is visible posteromedially.

**Remarks.** Gross [36] described some anterior lateral plates of smaller individuals as *R*. *kosorensis*. The plates are very similar in shape to the AL of *T*. *inopinatus*, but always lack the dermal ornament and other diagnostic features and are always found in isolation. Therefore, these finds (nos. NM Lc 3, 187 and counterpart 472, 465 and counterpart 466; [36], pl. 5, f. 2, textfig. 4B) must be left in open nomenclature.

**Genus *Sudaspis* gen. nov.** *urn*:*lsid*:*zoobank*.*org*:*act*:*4949C1DA-BFD6-4FAE-B85F-EFFED9517D8B*

(Fig 7)

**Fig 7:**
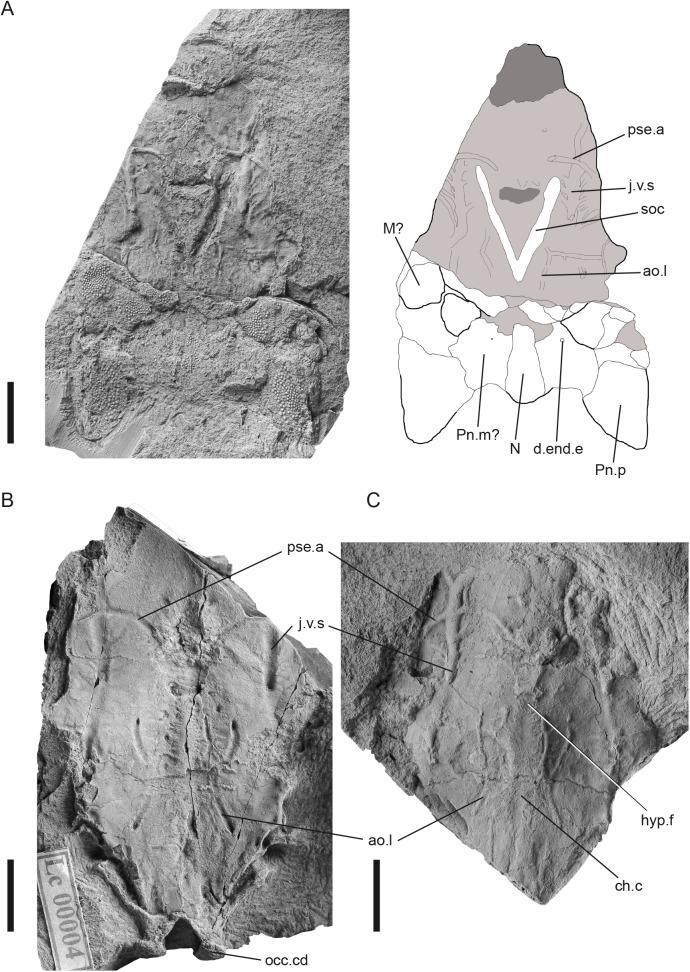
*Sudaspis chlupaci* gen. et sp. nov. (A), holotype, NM Lc 27, posterior part of skull roof attached to endocranium; (B), endocranial base, NM Lc 4; (C), endocranial base, NM Lc 496. Scale bars = 10 mm. Abbreviations: ao.l lateral aorta; ch.c chordal canal; d.end.e external foramen for the endolymphatic duct; hyp.f hypophyseal fenestra; j.v.s secondary jugular vein; M marginal plate, N nuchal plate; occ.cd occipital condyle; Pn.m medial paranuchal plate; Pn.p posterior paranuchal plate; pse.a efferent pseudobranchial artery; soc supraorbital sensory line groove.

**Diagnosis.** Jawed vertebrate possessing a broad, flat endocranium with an anterior prenasal expansion of the trabecular region. Prenasal region long. Suborbital shelf narrows abruptly at junction with prenasal region, creating distinct”shoulder” in endocranial margin. Endocranium tapering strongly posteriorly to a narrow glenoid region with prominent condyles. Grooves of lateral aortae on ventral face of endocranium narrow, shallow, together forming hourglass shape, branching anteriorly, ending anteriorly in two clearly visible foramina at level of anterior postorbital process and efferent hyoid artery. Endocranial surface between aorta grooves not rugose. Grooves of secondary jugular veins very obvious, wide, nearly straight, giving off several minor branches laterally, main mesial branch attached anterior to crossing of efferent pseudobranchial artery. Posterior part of skull roof relatively short. Large paranuchal plates form projecting posterior corners of skull roof. Dermal ornament composed of small, close-spaced, round tubercles without ridges.

**Derivation of name.** After the name of the street “V Sudech” (in barrels) in Prague-Radotín, which runs parallel to the quarries, its name reflecting the characteristic shape of the upper part of the profile resembling three barrels as well as the shape of the endocranium of *S*. *chlupaci*; and the Greek “aspis” (shield).

**Type and only known species.** *Sudaspis chlupaci* sp. nov.

***Sudaspis chlupaci* sp. nov.***urn*:*lsid*:*zoobank*.*org*:*act*:*386A13CD-82D5-412F-9B7E-D06015C508DF*

(Fig 7)

1959 *Radotina kosorensis* Gross; Gross [36]: pl. 2, fig. 6–7; pl. 3, fig. 5; pl. 4, fig. 1–2, textfig. 1F; 2C; –5.

1969 *Radotina kosorensis* Gross; Stensiö [47], fig. 105B.

**Diagnosis.** As for genus.

**Derivation of name.** In honour of late professor Ivo Chlupáč, a famous Czech invertebrate palaeontologist and stratigrapher, and a great enthusiast for early vertebrate research in the Czech Republic.

**Holotype.** Base of endocranium with posterior dermal skull roof cover. Figured by Gross [36], pl. 3, fig. 5, textfig. 1F. Housed in the Národní Muzeum in Prague with inventory number NM Lc 27 (Fig 7A).

**Type horizon.** Lower Devonian, Lochkovian; Lochkov Formation, Radotín Limestone.

**Type locality.** Černá rokle near Kosoř in Prague-Radotín.

**Material.** Endocrania NM Lc 4 (and counterpart NM Lc 32), NM Lc 469; endocranium with skull roof plates NM Lc 27.

**Description.** The holotype (Fig 7A) has the posterior part of the dermal skull roof preserved, but this is not easy to interpret. Prominent paranuchal plates, similar to those of *Romundina* [28], form the posterolateral corners of the roof. The nuchal appears to be narrow and small. It is certainly separated from the paranuchals, but it is not clear whether the intervening space is occupied by a single medial paranuchal (posterior central), as in *Romundina*, or by a mosaic of smaller bones. Anterior to the paranuchals, the region occupied in *Romundina* by marginals, anterior paranuchals and centrals [28] appears to consist of a greater number of smaller bones. A full interpretation of the skull roof must await the discovery of additional material, but it is already clear that the skull roof of *Sudaspis* is diagnostically different from those of both *Tlamaspis* and *Radotina*. The dermal ornament is composed of small, close-spaced, round tubercles without ridges.

The facial region of the endocranium is differently proportioned to *Romundina*, *Radotina* and *Tlamaspis*. As in *Tlamaspis*, the length of the facial region is just over 50% of the total endocranial length, contrasting with approximately 42% in *Romundina*, but in *Sudaspis* the extra length is due entirely to the elongated prenasal region; the proportions of the postnasal region are similar to *Romundina* [21] and *Radotina*. The suborbital shelf narrows abruptly at its anterior end, creating a distinct”shoulder” in the endocranial margin. Posteriorly, the endocranium tapers strongly to a narrow glenoid region with prominent condyles. The shallow, narrow, anteriorly branching grooves for the lateral aortae form an hourglass shape. The area between these grooves is smooth, not rugose as in *Tlamaspis*, but further anteriorly there is a low and narrow midline ridge flanked by numerous small transverse "scratches", that extends forwards towards the hypophysial region. The secondary jugular vein grooves are wide and nearly straight, branching laterally and mesially, the main mesial branch attaching anterior to the crossing with the canal of the efferent pseudobranchial artery. A transverse vascular groove running medially from the postorbital processes and crossing the jugular vein canal is interpreted as transmitting the efferent hyoidean artery. The posterior postorbital process is visible but difficult to interpret in the available material. Posterior to this process, the occipital region is both longer and more strongly tapering than in *Tlamaspis*. It shows a curious double margin, with an external crista on each side that posteriorly joins with the condylar region of the occiput. This clearly reflects an anatomical feature, but it is not possible to say whether it retains its natural shape or has been modified by dorsoventral compression.

Order and Family indet.

**Genus *Holopetalichthys* von Koenen**, **1895**

(Fig 8)

**Fig 8:**
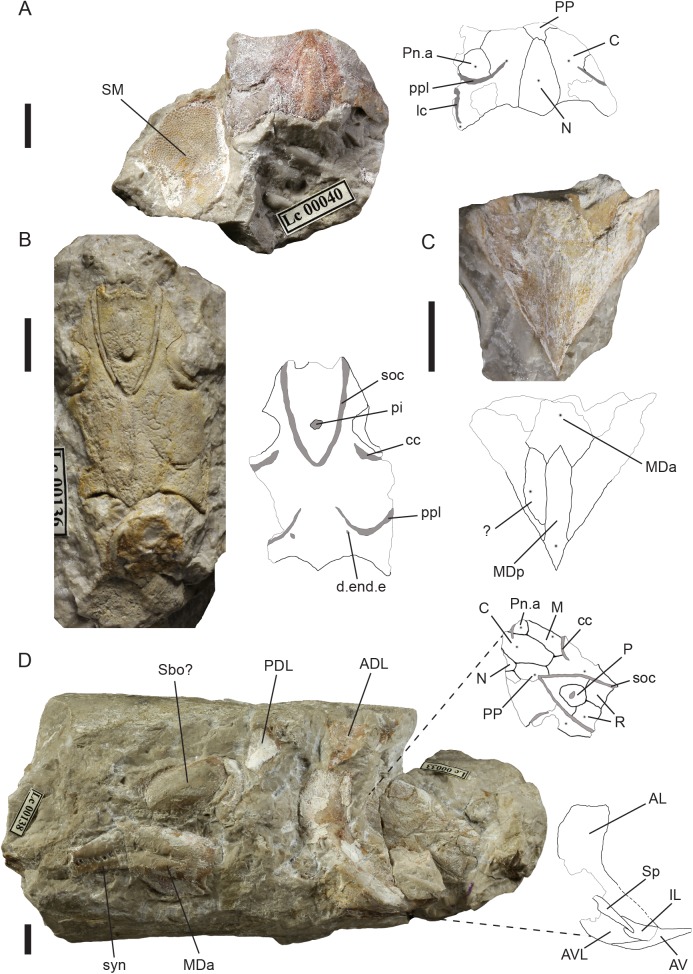
*Holopetalichthys primus* (Barrande, 1872). (A), holotype, NM Lc 40, posterior part of the skull roof, inner cast of submarginal plate; (B), skull roof, NM Lc 136; (C), median dorsal plate complex, NM Lc 139 (D), head and thoracic shield, NM Lc 33 and 138. Scale bars = 10 mm. Abbreviations: ADL anterior dorsolateral plate; AL anterior lateral plate; AV anterior ventral plate; AVL anterior ventrolateral plate; C central plate; cc central sensory line groove; d.end.e external foramen for the endolymphatic duct; IL interolateral plate; lc lateral sensory line groove; M marginal plate; MDa anterior median dorsal plate; MDp posterior median dorsal plate; N nuchal plate; P pineal plate; PDL posterior dorsolateral plate; pi pineal organ; Pn.a anterior paranuchal plate; PP postpineal plate; ppl posterior pitline; R rostral plate; Sbo suborbital plate; SM submarginal plate; soc supraorbital sensory line groove; Sp spinal plate; syn synarcuale.

**Diagnosis.** Jawed vertebrate with perichondrally ossified endocranium. Endocranium with long preorbital region incorporating fused nasal capsule. Dermal skull roof entirely macromeric, tesserae absent. Supraorbital and preorbital margin of skull roof formed by single elongate supraorbital ossification. Paired rostral plates. Posterior end of postpineal plate contacts nuchal plate, separating the central plates in the midline. Central plate elongated, reaches posterior margin of skull roof. The tubercles of the dermal ornament round, composed mainly of bone. Median dorsal plate complex composed of a spine-shaped posterior median dorsal, a broad butterfly-shaped anterior median dorsal, and a posterolaterally positioned plate.

**Type and only known species.** *Holopetalichthys primus* (Barrande, 1872)

**Remarks.** The specimens described herein as the genus *Holopetalichthys* had originally been split into three separate species. Barrande [32] erected the species *Coccosteus primus* based on specimen NM Lc 40 (Fig 8A). Its generic identification was challenged by Bayer [54]. Specimen NM Lc 136 (Fig 8B) was described by von Koenen [33] as a monospecific new genus and named *Holopetalichthys novaki*. Gross [35], without knowledge of the published specimens, described NM Lc 33 (Fig 8D) as a new species of the genus *Radotina* with the species name *tuberculata*. Westoll [37] was the first to note the common characters of NM Lc 40 and 33 and united them in a single species using the name *Radotina prima*. He never mentioned NM Lc 136 or the generic name *Holopetalichthys*. Ørvig [40] pointed out this fact of priority, but suggested to dismiss the older name as a nomen nudum. Although Denison [25] repeated this suggestion, none of them applied to officially accept the younger name *Radotina*. However, this would not be possible, as the name *Holopetalichtys* had been widely used in contemporary literature (e.g. [48]; [41] and references therein). Goujet [10] doubted the determination of *R*. *prima* to the genus *Radotina*, based on the absence of tesserae and the premedian plate, which has been added to the reconstruction by Denison ([25] figure 22C) without supporting evidence. According to Goujet [10] the differences in histology and the shape of the dermal sculpture are strong arguments to exclude the species *R*. *prima* from the order Acanthothoraci. However, he did not suggest any taxonomic or systematic solution.

Our examination of the material historically assigned to *Coccosteus primus*, *Holopetalichthys novaki* and *Radotina tuberculata* shows that it represents a single species, which is so different from *Radotina kosorensis* and *R*. *tesellata* that assignation to *Radotina* is out of the question. It is also very different from *Coccosteus*. We establish the combination *Holopetalichthys primus* for this species. It has already been used by Chlupáč [55,56], with the nomination of a lectotype (NM Lc 40, Barrande’s holotype of *Coccosteus primus*), but without a formal diagnosis; we present a diagnosis to complete the taxonomic process. The assignation of *Holopetalichthys* to the Acanthothoraci must be regarded as doubtful, given that there is no evidence for a projecting prenasal area or a premedian plate.

Zhu and Wang [57] described a new species of the order Macropetalichthyidae named *Holopetalichthys longhuaensis*. Their diagnosis is not in accordance with the former description [33], which is not even cited in the paper, and the genus *Holopetalichthys* is considered a genus novum. Therefore *Holopetalichthys longhuaensis* must be considered a preoccupied taxon and thus the determination by Zhu and Wang is invalid.

***Holopetalichthys primus* (Barrande**, **1872)**

(Fig 8)

1872 *Coccosteus primus*. Barr.; Barrande [32], pp. 640–641, pl. 29–1, 2.

1895 *Holopetalichthys Novaki* v. Koenen; von Koenen [33], pp. 25–27, pl. IV, 2 a, b.

1905 *Coccosteus primus* Barr. (“doubtfully belonging to genus *Coccosteus”*); Bayer [54], p. 29.

1905 *Holopetalichthys Novaki* Koen; Bayer [54], p. 33.

1950 *Radotina* sp. Gross; Gross [34], pp. 113–118, fig. 1B.

1958 *Radotina tuberculata* n. sp; Gross [35], pp. 21–23, pl. 1, fig. 3; pl. 3, fig. 5–6; textfig. 6.

1967 *Radotina prima* (Barrande); Westoll [37], pp. 83–88, fig. 2B.

1969 *Radotina tuberculata* Gross; Stensiö [47], fig. 104A.

1971 *Holopetalichthys kosorensis*; Moy-Thomas & Miles [48], fig. 8.18A.

1975 *Radotina tuberculata* Gross; Ørvig [40], pp. 48, 49, 66, fig. 1D.

1978 *Radotina prima* (BARRANDE); Denison [25], pp. 36, 79, fig. 1B, 22C.

1984 „*Radotina” prima*; Goujet [10], pp. 224, 228, 230, 235, 238, 239, fig. 8B, 9, 12.

1996 '*Radotina' prima*; Janvier [49], p. 170, fig. 4.57D.

2002 *Coccosteus primus* Barr. = *Holopetalichthys primus* (Barrande, 1872); Chlupáč [55], explication to pl. 29, fig. 1, 2 (no page number).

2002 *Holopetalichthys primus;* Chlupáč et al. [56], fig. 83, 2.

2009 *Radotina prima* (Barrande); Vaškaninová [51], p. 195.

2011 “*Radotina*” *prima* (Barrande, 1872); Dupret et al. [29], fig. 3C, pp. 531–532, 537.

2011 *Holopetalichthys primus* (Barrande, 1872); Vaškaninová [38], p. 52.

**Diagnosis.** As for genus.

**Holotype.** The posterior part of the skull roof with associated submarginal plate. Figured by Barrande [32], pl. 29–1, 2. Housed in the NM with the catalogue number NM Lc 40 (Fig 8A).

**Type horizon.** Lower Devonian, Pragian; Prague Formation, upper Koněprusy Limestone.

**Type locality.** Koněprusy near Beroun, south-east of Prague, probably the area of the present day Houba Quarry.

**Material.** Head and associated thoracic region NM Lc 33+NM Lc138 (ČF 5 of Westoll [37]); skull roof NM Lc 40 (ČF 6 of Westoll [37]), NM Lc 136; cheek plate NM Lc 145; median dorsal plate NM Lc 139 (ČF 2 of Westoll [37]), NM Lc 141 (ČF 1 of Westoll [37]); thoracic shield plates NM Lc 134 (ČF 3 of Westoll [37]) and counterpart NM Lc 135 (ČF 4 of Westoll [37]).

**Remarks.** Gross [35] described only one specimen, NM Lc 33 ([35], pl. 1, f. 3, textfig. 6A), as *Radotina tuberculata*. It was known as an anterior part of the skull roof; its posterior part was thought to be lost after deposition, as glue marks were still visible on the fracture surface. We have discovered that NM Lc 33 is a part broken off specimen NM Lc 138, which contains the thoracic region and a synarcual ossification (described but not figured by Westoll [37]). The two pieces had already become separated by the 1950s when Gross started to study the material.

Specimen HMN f 633 in the Humboldt Museum für Naturkunde, Berlin, from the Lower Devonian Taunus Quartzite near Rüdesheim, Rheinish Slate Massif, Germany, is identifiable as the anterior right side of the skull roof of *Holopetalichthys*. It was decribed as *Radotina* sp. by Gross [35]. Although its attribution to *Holopetalichthys* is unproblematic, its incompleteness, together with the fact that it is much larger than the Koněprusy specimens and derives from another locality, means that we cannot confidently identify it as the species *Holopetalichthys primus*. We provisionally designate it *Holopetalichthys* sp.

**Description.** The skull roof is composed of macromeric dermal plates, tesserae are absent. The plates are clearly recognisable by their ossification centres visible in the vascular middle layer (Fig 8A, 8C and 8D). The nostrils are positioned anterior to paired rostral plates and face anterodorsally. The skull roof probably terminated at this point, as we have no record of a premedian plate. The rostronasal capsule was clearly not loosely attached as in *Romundina* or *Tlamaspis*, where it tends to become detached from the rest of the endocranium.

The dorsal rim of the orbit carries a narrow, sharply delineated encircling groove, lying close to the orbital margin anteriorly and posteriorly but diverging away mesially from it in the middle (Fig 8B). The preorbital and supraorbital area is formed by a single elongate supraorbital ossification bounded by the supraorbital sensory line mesially, by a suture with the central plate posteriorly, and by the central sensory line groove posterolaterally (Fig 8D). The central sensory line runs towards the ossification centre of the central plate. The paired rostral plates are bounded by the supraorbital sensory lines laterally, and by a suture with the round pineal plate posteromesially. In the pineal area medially to the supraorbital sensory lines lies the pineal plate. The pineal organ was originally positioned in the ossification centre of this plate, but there is no pineal opening.

Posterior to the central sensory line the ossification centres of another two plates are recognisable—the marginal and anterior paranuchal plates [37]. The marginal plate extends posteriorly separating the anterior paranuchal plate from the skull roof margin (Fig 8A and 8D). The anterior and posterior paranuchal plates are separated by the posterior pit lines, but the posterior paranuchal is not well preserved.

The central plates are elongated, oval, wider anteriorly, and do not meet mesially. The obliquely oriented posterior pit lines extend towards the ossification centres of the plates in NM Lc33 and NM Lc 40; in NM Lc 136 they appear to cross the ossification centres and continue towards the posterior meeting point of the suborbital canals. The centrals are separated in the midline by the nuchal plate and the elongated postpineal plate. The supraorbital sensory lines meet close to the ossification centre of the postpineal plate. The nuchal plate is relatively short and widens posteriorly, terminating in a blunt midline point. The sensory lines occupy deep grooves, recessed into to the dorsal surface of the endocranium. The matrix fill of the canal itself is preserved in some places, notably in the supraorbital canals of NM Lc 136 (Fig 8B); it is surrounded by an extremely thin mineralized boundary layer that appears to represent the membranous walls of the canal. Sensory lines may follow sutures (as in the case of the supraorbital canal following the suture between the rostral and supraorbital plates), but their ends always point towards the ossification centres of bones. The endolymphatic duct opening is clearly visible on the specimen NM Lc 136 (Fig 8B).

Only one plate from the cheek area is known, the submarginal plate preserved as an imprint on the specimen NM Lc 40 (slightly displaced posteriorly; Fig 8A). It has a typical shape for this bone, widening anteriorly with an oval projection dorsally. A fragment of similar submarginal plate occurs also on the specimen NM Lc 145.

The median dorsal plate complex, which at first sight might be interpreted as a single bone, is in fact composed of a spine-shaped posterior median dorsal, a broad butterfly-shaped anterior median dorsal and an un-named posterolaterally positioned plate. The posterior dorsal has a triangular cross section, with a dorsal crest ornamented by oblong ridges (Fig 8C). Specimen NM Lc 138 (ČF 5 of Westoll [37]; Fig 8D) displays a complete thoracic complex preserved in 3D along with isolated trunk shield plates. Overall, the thoracic armour is very short anteroposteriorly, taking the form of a narrow shoulder girdle, somewhat as in acanthothoracids and ptyctodonts. The damaged anterior lateral plate resembles the shape of the AL plates found in *Tlamaspis inopinatus* or *Palaeacanthaspis vasta* [22]. Anteroventrally it appears to taper to a point. The anteroventral ramus of the AL is bounded ventrally by a spinal plate, which carries a short posterolaterally directed pectoral spine, and anterior to the spinal plate an interolateral plate with a forked posterior end that clasps the spinal plate. Remarkably, the area ventral to the spinal and interolateral plates is occupied not by a single anterior ventrolateral plate, but by two plates. We tentatively interpret them as the anterior ventral and anterior ventrolateral plates (AV, AVL; Fig 8D), resembling the condition reconstructed in *Romundina* cf. *stellina* [28]. The AL plate is ornamented with badly preserved tubercles in the mesial area and on the postbranchial lamina. Dorsally to the AL plate lie two smaller isolated plates, probably the anterior and posterior dosolateral plates. The median dorsal plate complex has the dorsal spine broken off and its inner bone layer is exposed along with the spine cavity (compare the preservation of *P*. *vasta* [22]). The median dorsal plate is connected to a robust bony element in the area of the former dorsal spine, the synarcual [37], which appears to be slightly displaced. The synarcual has a joint surface on the posterior end. Medially, there is a band of small openings—the accreted vertebrae. In the vicinity of the MD plate lies a larger, oval plate, probably a displaced suborbital plate.

The surface sculpture of specimen NM Lc 136 (Fig 8B) is mostly abraded, broken off or destroyed by polishing. Specimen NM Lc 33, also strongly polished, has the tubercles most clearly visible on the rostral plates. They are poorly preserved, but they evidently were rather bulky, not star shaped or striated. Similar tubercles are very well preserved in the imprint of the submarginal plate of the holotype (Fig 8A) and have been examined on a latex peel. They seem to be built mostly of a vascular bone layer, the semidentine is restricted only to the top. This type of tubercle occurs also on the probable spinal plate of the specimen NM Lc 134, which was found in the same piece of rock as the imprint of a median dorsal plate complex of *Holopetalichthys*. The counterpart of the MD complex is found on specimen NM Lc 135. Median dorsal plate complexes of the same shape can be found on specimens NM Lc 139 (Fig 8C) and NM Lc 141; both have a distinctive dermal ornament. Specimen NM Lc 136 (Fig 8B) has a fragment of another plate attached to the posterior border of the skull roof. Its position suggests that it may be part of a median dorsal complex, but it is too incomplete to identify.

## Discussion

### Phylogenetic and evolutionary implications

We do not present a phylogenetic analysis in this paper because it would be premature at this stage. Detailed anatomical investigations of all the Lochkovian and Pragian placoderms of the Prague Basin are under way, on the basis of synchrotron microtomography, and will produce large amounts of new phylogenetic data within the next few years. However, our taxonomic findings already have a major impact on the phylogenetic significance of the Prague Basin placoderms, because they show that past interpretations have been based on multi-taxon chimaeras. This is especially important because they have been widely recognized as very primitive placoderms, potentially informative about questions such as the origin of a macromeric skeleton. For example, Moy-Thomas and Miles [48] consider at some length the significance of dermal bone variability in "*Holopetalichthys kosorensis*", which is described (following [37]) as having a skull roof that shows individual variation between fully macromeric and largely tesselated conditions. But as we have shown above, this "*Holopetalichthys kosorensis*" is a multi-taxon composite that includes aspects of *Radotina kosorensis*, *Tlamaspis inopinatus* and *Holopetalichthys primus*, three very different placoderms, each with its own characteristic (and apparently stable) skull roof pattern. The extreme individual variability posited by Westoll [37] and Moy-Thomas and Miles [48] simply does not exist. Given this unusual situation, with its potential for seriously corrupting phylogenetic and evolutionary data sets relating to early gnathostomes, we urge that all previous descriptions and interpretations of the Prague Basin placoderms be excluded from the current discussion of these questions.

Our taxonomic revision reveals that the Early Devonian of the Prague Basin contains a remarkable richness of acanthothoracids, especially in the Lochkovian where the placoderm fauna can now be seen to consist of at least four acanthothoracids (*Radotina kosorensis*, *Tlamaspis inopinatus*, *Sudaspis chlupaci* and *Kosoraspis peckai*) with no other placoderm groups known to be represented in the assemblage. This is interesting from an ecological perspective (see below) but also with regard to the evolution of dermal bone macromery.

The last few years have seen a remarkable about-turn as regards the evolution of macromery, from an almost universally held belief that the macromeric skeletons of placoderms and osteichthyans are independently evolved (e.g. [14,49]) to widespread acceptance that they are homologous (e.g. [6,13]). This extends the homologue set of our own macromeric skeleton down into the upper part of the gnathostome stem group, with the implication that the absence of homologous bones in chondrichthyans is secondary [13]. The lower part of the gnathostome stem group comprises jawless bony vertebrates known as ostracoderms. Among these, thelodonts and anaspids are entirely covered with scales, and are thus micromeric in the same sense as chondrichthyans. Galeaspids and osteostracans have dermal head shields that are normally solid one-piece structures in the adult, but are composed of tesserae that fuse late in ontogeny [58,59]. This can also be considered as a type of micromeric skeleton, because the tesserae do not appear to have conserved individual identities, but in osteostracans at least the anterior rim of the head shield is composed of larger, morphologically distinctive bones [59]. Heterostracans have macromeric dermal headshields, but these show no pattern similarities with either placoderms or osteichthyans [49]. However, bands of tesserae are sometimes present between the macromeric bones (notably in the psammosteid *Drepanaspis* [49]), in a manner very reminiscent of *Radotina*.

The lack of obvious homologies between the macromeric patterns of heterostracans and placoderms suggests that the macromeric bones that characterize placoderms and osteichthyans evolved de novo within the gnathostome stem group, at approximately the same time as the origin of jaws. However, the head-shield tesserae of osteostracans, galeaspids and some heterostracans are strongly reminiscent of those in acanthothoracid placoderms such as *Radotina*, raising the intriguing possibility that their presence in acanthothoracids could be a primitive character retained from agnathan ancestors. The fact that many recent phylogenetic analyses recover acanthothoracids as among the most basal jawed vertebrates [7,8,13,19] makes this hypothesis all the more interesting.

Given our limited understanding of the skull roof patterns in some Prague Basin placoderms, especially *Sudaspis*, and the current lack of microanatomical data for the tesselated regions, it is too early to venture on a detailed interpretation of these patterns and their significance. However, one striking observation that is worth highlighting is the relationship between the premedian plates of *Radotina kosorensis* and *Romundina stellina*. *Romundina* has a unitary premedian plate without obvious sutures, but synchrotron microtomography reveals that its internal vasculature divides into two discrete domains ([20], figures 1C and 2B). The thick anterior part of the plate contains several superimposed layers of internal vasculature, all with a radial pattern originating at a midline point near the anterior margin. By contrast, the much thinner posterior half of the plate has a non-radial vasculature with a largely anteroposterior orientation, principally occupying the contact surface between the dermal bone and the underlying endoskeleton. In *Radotina kosorensis* (Fig 2A), the premedian dermal bone cover consists of two discrete components: a premedian plate anteriorly and a field of tesserae posteriorly. The premedian plate shows a clear radial structure, originating from the same place in the anterior midline as the radial vascular pattern of *Romundina*. This suggests that the seemingly unitary premedian plate of *Romundina* incorporates the same combination of an anterior plate and a posterior field of tesserae as the premedian dermal skeleton of *Radotina*, but that the sutures have become obscured. Further data on dermal bone microanatomy, which should emerge from the synchrotron microtomography investigation of the Prague Basin acanthothoracids, will provide a more robust basis for future hypotheses about the evolution of macromery.

### Taphonomy

The large number of morphologically diverse and well preserved placoderm specimens from the localities Černá rokle (Lochkovian) and Koněprusy (Pragian) provides a good database for studies of taphonomy and subsequent diagenetic effects. The strongly dorsoventrally flattened skulls with partly exposed endocranial structures represent the best preserved specimens from the Černá rokle quarries. In life, the endocrania were built of unmineralized cartilage, covered by a thin layer of perichondral bone; after death the cartilage decayed and was replaced with sedimentary infill. The endocrania are flattened to a thickness of only a few millimetres [36], contrasting with the three-dimensional preservation of similar endocrania of *Romundina* in the Lochkovian of Prince of Wales Island [7,28,40] and *Radotina tesselata* in the Pragian Koněprusy limestone (see below). The endocrania display openings and imprints of nerves and blood vessels. The slabs containing the specimens were usually split in the horizontal plane, so the inner cast of the specimen represents the base of the endocranium, i.e. the inner ventral surface. Although the compression is strong, some prominent features (e.g. the prenasal area of *T*. *inopinatus*) have retained their threedimensionality. No endocranium is laterally compressed, even in semi-articulated specimens where the adjacent shoulder girdle is preserved in lateral view (Fig 6). This probably reflects the fact that acanthothoracid endocrania, like those of most placoderms, were rather broad and flat even in life (e.g. [21]) and thus tended to settle in a horizontal position in the sediment.

In most cases, the dorsal endocranial surface is covered by at least the central and nuchal plates of the skull roof. The cheek plates (e.g. the submarginal) are preserved separately. They were not attached to the endocranium as firmly as the skull roof plates and are found associated to the endocranium or the thoracic plates with their ventral surfaces exposed. Also the anterior lateral plates are preserved isolated with the strongly compressed scapulocoracoid always firmly attached. Other thoracic plates are found isolated as well; the median dorsal plate has the spine broken off as a rule.

A number of specimens of *Tlamaspis inopinatus* show a specific form of preservation where the specimen, despite being flattened, is complete with the head closely associated to the shoulder girdle and the visceral skeleton pushed from beneath the head laterally (Fig 6A and 6B). The process can be reconstructed as a dorsoventral or semi-lateral compression of a complete anterior body of the fish, e.g. head and thoracic shield. The ventral thoracic plates are unknown; the complete posterior body has not yet been discovered. Some specimens show scattered disarticulated body scales associated to separate dermal plates or their fragments.

The postmortem transport of placoderms found in the Černá rokle quarries was apparently only over a short distance. The model of the prevailing taphonomic processes can be reconstructed from the patterns of fragment distribution. The carcasses were being transported to the anoxic basin from a supposed platform by turbidite currents. During the process of decay, before the specimens were covered by sediment, another short distance transport of smaller and lighter parts (e.g. fragments of dermal plates or scales) may have occurred. The strong compression was a part of the subsequent diagenetic process. However, the accompanying fauna, including other fish (e.g. fin spines of *Machaeracanthus* [44]), does not display such strong compression [41]. In rare cases the specimens display a slight horizontal displacement of the dermal plates (e.g. NM Lc 29; Fig 4B). The overlap increases but the plate position is constant.

The Koněprusy material is preserved three-dimensionally. The most common specimens are skull roofs along with median dorsal plate complexes. The dermal plates are split through their middle vascular bone layer; the surface sculpture was preserved in the missing counterparts, but can be reconstructed from a few areas of natural mould present on the specimens. The recently associated specimens NM Lc 33 and NM Lc 138 (Fig 8D) display a fully preserved head situated inside the thoracic region with adjacent separate plates and vertebral column, all deposited within the infill of an orthocone cephalopod shell.

### Palaeoecology

The Černá rokle quarries yielded the most abundant placoderm material, greatly exceeding the number of specimens from the remaining localities of the Prague Basin [31]. It is uncertain whether this abundance is a taphonomic effect, the result of intensive collecting of all fragments during the period of active quarrying, or if the placoderms were genuinely more abundant in the Lochkovian of the Prague Basin. Perner [42] and Gross [36] supposed the existence of a "fish bearing" layer or accumulation exposed at the time of active quarrying, nowadays inaccessible, but probably belonging to a relatively short overlap of the *Paranowakia bohemica* and *P*. *intermedia* tentaculite zones (for more details see [31]). Only scarce fragments of placoderm fossils can be found nowadays. *Contra* King et al. [12], who argue that phylogenetically basal placoderms were benthic, we suggest that the Lochkovian acanthothoracids were most probably nektonic active swimmers, considering the shape of their crania and trunk armours, and the lateral position of the orbits [25, 28]. The environment in the Lochkovian of the Černá rokle is supposed to be relatively deep water [43].

In the bioherm environment of the Koněprusy quarries in the Pragian the small placoderms may represent prey for larger predators (e.g. the acanthodian genus *Machaeracanthus* found in the same strata [44]). Often the placoderm remains are preserved in the infill of large orthoconic cephalophod shells (e.g. Fig 8B and 8D). The prominence of acanthothoracids in these assemblages is remarkable, especially in the Lochkovian Radotín Limestone where at least four morphologically divergent acanthothoracids (*Radotina*, *Tlamaspis*, *Sudaspis* and *Kosoraspis*) occur side by side. We are not aware of another example of such an acanthothoracid-dominated placoderm fauna. By contrast, Emsian and later placoderm assemblages from the Prague Basin contain only arthrodires [31].

## Conclusions

This paper has significantly changed the view of the systematic structure of the Prague Basin Lower Devonian (Lochkovian and Pragian) placoderm fauna, finally resolving the long prevailing confusion about the variable character states in individual taxa. Four genera (*Radotina*, *Tlamaspis*, *Sudaspis* and *Holopetalichthys*) have been established and provided with unambiguous diagnoses based on well documented unique characters. The species name *Radotina kosorensis* Gross, 1950 is now limited to a few specimens that share unique characters with the holotype. *Radotina tesselata* Gross, 1958 remains a single specimen species. *Radotina* has a skull roof with large tesselated areas separating the macromeric bones. *Tlamaspis* is characterised by a very long facial region and a dermal skull roof lacking tesserae. *Sudaspis* has a long prenasal region with a distinctive, stepped lateral profile. The skull roof is essentially macromeric, including large and prominent paranuchals. We consider all the previously published attempts to reconstruct or interpret the “radotinid” material, apart from references to holotypes, as invalid because they inadvertently combine information from more than one taxon.

We have also resolved the taxonomic confusion following the generic determination of the so-called “*Radotina*” *prima* (previously known as *Coccosteus primus*, *Holopetalichthys novaki* and *Radotina tuberculata*) and its problematic position within the acanthothoracids. We establish the name combination *Holopetalichthys primus* (Barrande, 1872) for a number of specimens displaying the same unique characters. The genus *Holopetalichthys* is excluded from the acanthothoracids on the basis of the probable absence of the prenasal area as well as other distinct characters listed above. However, it displays many unusual features in its dermal skeleton. We interpret *Holopetalichthys* as a basal placoderm of uncertain affinities.
